# Molecular Characterization of *Campylobacter jejuni* Clones: A Basis for Epidemiologic Investigation

**DOI:** 10.3201/eid0809.02-0122

**Published:** 2002-09

**Authors:** Kate E. Dingle, Frances M. Colles, Roisin Ure, Jaap A. Wagenaar, Birgitta Duim, Frederick J. Bolton, Andrew J. Fox, David R.A. Wareing, Martin C.J. Maiden

**Affiliations:** * University of Oxford, Oxford, United Kingdom; †Public Health Laboratory, Royal Preston Hospital, Preston, United Kingdom; ‡ Institute for Animal Science and Health, Lelystad, the Netherlands; § Public Health Laboratory, Withington Hospital, Manchester, United Kingdom

**Keywords:** *Campylobacter jejuni*, multilocus sequence typing, clonal complex, molecular epidemiology

## Abstract

A total of 814 isolates of the foodborne pathogen *Campylobacter jejuni* were characterized by multilocus sequence typing (MLST) and analysis of the variation of two cell-surface components: the heat-stable (HS) serotyping antigen and the flagella protein FlaA short variable region (SVR). We identified 379 combinations of the MLST loci (sequence types) and 215 combinations of the cell-surface components among these isolates, which had been obtained from human disease, animals, food, and the environment. Despite this diversity, 748 (92%) of the isolates belonged to one of 17 clonal complexes, 6 of which contained many (318, 63%) of the human disease isolates. Several clonal complexes exhibited associations with isolation source or particular cell-surface components; however, the latter were poorly predictive of clonal complex. These data demonstrate that the clonal complex, as defined by MLST, is an epidemiologically relevant unit for both long and short-term investigations of *C. jejuni* epidemiology.

*Campylobacter jejuni* is the most frequently reported cause of acute inflammatory gastroenteritis in industrialized countries and a major cause of intestinal disease in children <2 years of age in developing countries (1,2). *C. jejuni* infection is widely perceived as an increasing problem; for example, the number of reported cases of *C. jejuni*-associated enteritis increased sixfold in the United Kingdom from 1977 to 2000 (3,4). The syndromes associated with *C. jejuni* infection range from mild enteritis to severe invasive disease, and sequelae can occur, including the autoimmune-mediated demyelinating neuropathies Guillain-Barré and Miller Fisher syndromes (5). The intestines of many feral and commercially reared birds, livestock, domestic pets, and animals are asymptomatically colonized by this bacterium, which is widely distributed in the environment and food. Transmission to humans is thought to occur through food, drinking water, and pets (6,7).

 Elucidation of the epidemiology of this zoonosis is complicated by the sporadic nature of the disease (8), along with the organism’s wide distribution, high levels of genetic and antigenic diversity, and a lack of representative population samples (9). The absence of precise information on the relative importance of different sources of human infection has hindered the development of effective disease-control measures and is a major challenge in preventing human disease. The characterization of *C. jejuni* isolates has relied on serologic typing schemes since the 1980s (10,11), but the data available from these techniques have not resolved the epidemiology of human disease. Consequently, the development of a method for the characterization of *C. jejuni* isolates that is accurate, reproducible, and comparable among laboratories has been a research priority for many years (9). While numerous phenotyping and genotyping techniques that discriminate among isolates for short-term epidemiology have been developed, generating data that are comparable among different laboratories and that accurately identify relationships among isolates from diverse sources has proven more challenging.

 A multilocus sequence typing (MLST) scheme (12) has been developed and validated for *C. jejuni* (13). This approach, which exploits recent technical developments in high-throughput nucleotide sequence determination and combines them with conceptual advances in bacterial population biology, has proved to be successful for a number of bacteria (12–15). MLST is especially suitable for the investigation of diverse bacterial populations that have weakly clonal population structures (13,16,17). In common with multilocus enzyme electrophoresis, MLST indexes variation in housekeeping genes, which evolve slowly as they are under stabilizing selection for the conservation of metabolic function. The use of nucleotide sequence data directly accesses the variation in the targeted gene, and the technology employed is readily disseminated and highly reproducible (18). Further advantages are that the data are electronically portable, enabling comparison of isolates via the Internet without exchange of biological materials, and amenable to analysis by phylogenetic and population genetic techniques (19,20).

Initial analysis of *C. jejuni* populations with MLST confirmed the diverse genetic nature of the *C. jejuni* species and indicated that the population structure was likely to be weakly clonal (13,17). Weakly clonal bacterial populations comprise a number of clonal complexes, which correspond to “lineages,” i.e., groups of organisms presumed to derive from a common progenitor. However, the phylogenetic relationships among distinct complexes cannot in general be reconstructed because they have been disrupted by lateral gene transfer (20). The MLST approach has been used to demonstrate that a number of *C. jejuni* clonal complexes are associated with human disease and that isolates from several of these caused demyelinating neuropathies (13,21).

 We examined the relationships of *C. jejuni* isolated from a broad range of sources in two countries by MLST, which was used to define clonal complexes. The results of this analysis were compared with variation observed in two cell-surface components that have been previously used for bacterial typing: the heat-stable (HS) serotyping antigen, which was investigated serologically; and the flagella, which was investigated by nucleotide sequencing of the *flaA* gene short variable region (SVR). We also examined the extent to which different isolation sources and cell-surface component variants correlated with clonal complex.

## Methods

 A diverse collection of *C. jejuni* isolates, mainly from the United Kingdom and the Netherlands, was investigated. Most of these isolates, which originated from human disease, food animals or products, and an environmental source (Table 1), had been characterized by HS antigen serotyping (11,22–24). For each isolate, chromosomal DNA was prepared, and MLST was performed with seven housekeeping genes as described (13). The data were deposited in the *Campylobacter* Internet-accessible database, enabling the sequence types (STs) of the isolates to be established (http://campylobacter.mlst.net/). In addition, the nucleotide sequence of a 321-bp region of the *flaA* gene was determined for each isolate. This sequence encompassed the SVR (25) extending from *flaA* nucleotide positions 283–603 inclusive (FlaA amino acids 95–201). Primers FLA4F (5´-GGA TTT CGT ATT AAC ACA AAT GGT GC-3´) and FLA1728 (5´-CTG TAG TAA TCT TAA AAC ATT TTG-3´) or FLA625RU (CAA G[AT]C CTG TTC C[AT]A CTG AAG-3´) were used (25). The polymerase chain reaction amplification conditions were as follows: denaturation at 94ºC for 2 min, primer annealing at 50ºC for 1 min, and extension at 72ºC for 2 min for 35 cycles. Nucleotide sequence extension reactions were performed with primers FLA242FU (5´-CTA TGG ATG AGC AAT T[AT]A AAA T-3´) and FLA625RU, and BigDyeTM Ready Reaction Mix (Version 2, PE Biosystems, Foster City, CA) was used in accordance with the manufacturer’s instructions. Reactions products were separated with an ABI prism 3700 automated DNA sequencer (PE Biosystems). The peptide sequences encoded by the SVR nucleotide sequences were deduced, and each unique amino acid sequence was assigned FlaA SVR amino acid variant number in the order of discovery. The amino acid sequence of the FlaA SVR was used to provide an indication of the phenotypic diversity of a variable cell-surface component that is known to be subject to antigenic variation. The sequences were deposited in an Internet–accessible database of FlaA SVR sequences (http://outbreak.ceid.ox.ac.uk/campylobacter/).

**Table 1:** Sources of *Cambylobacter jejuni* isolates

Source	Country of Origin				Total
	United Kingdom	Netherlands	Others	Unspecified	
Human disease	370	79	25	27	501
Chickens	53	77	4	3	137
Lamb/offal	72	-	-	-	72
Beef cattle/offal	48	2	1	1	52
Sand from beaches	52	-	-	-	52
Total	595	158	30	31	814

 The initial step in the assignment of the STs to clonal complexes was to identify a founder or central genotype for each clonal complex. Founder STs were identified with the aid of heuristic methods such as the BURST algorithm and UPGMA cluster analysis, implemented in the computer program START (26), and split decomposition, implemented in the program SPLITSTREE (27). After the central genotypes had been identified, all remaining STs were assigned sequentially to clonal complexes. First, the STs differing at one locus from a founder were assigned, followed by those differing at two loci and finally those that differed at three loci. The clonal complexes were named after the ST of the central genotype, e.g., ST-21 complex (13). Assignment of STs, clonal complex, serotype, and FlaA SVR type permitted comparisons of these characters and their combinations with source of isolation.

 For each of the clonal complexes, the significance of any association with serotype, FlaA SVR, and isolation source was independently assessed by chi square test. For the cell-surface components, the distribution of individual variants expected if they were distributed randomly among the clonal complexes was calculated. This null hypothesis was compared with the observed distribution, and the statistical significance of any observed associations was tested. For the source of isolation, we tested the null hypothesis that the distribution of clonal complexes among isolates from each source was the same as the distribution of clonal complexes found in human disease isolates.

## Results

### *C. jejuni* Clonal Complexes

 The collection of 814 isolates contained 379 distinct sequence types. On the basis of their STs, 748 (92%) of the isolates were assigned to 1 of 17 clonal complexes (Table 2) comprising >6 members, with 318 (63%) of the 501 isolates obtained from human disease being assigned to 1 of 6 clonal complexes (ST-21, ST-45, ST-206, ST-61, ST-48, and ST-257 complexes). The STs of the remaining 66 isolates occurred either once (31 isolates, 3.8%) or in combinations of two to four identical or related sequence types (35 isolates, 4.2%). These included seven reference isolates for the Penner serotyping scheme (11) and eight isolates with one to six alleles that had divergent nucleotide sequences.

**Table 2:** Clonal complexes of *Campylobacter jejuni* isolates

Clonal complex	Isolates (n)	ST^a^ of central genotype							No. of STs	No. of cell-surface component variants			Cell-surface component variants (p<0.001) ^c^	
		*^aspA^*	*^glnA^*	*^gltA^*	*^glyA^*	*^pgm^*	*^tkt^*	*^uncA^*		HS serotypes^b^	FlaA SVR	Antigen combinations	HS serotypes	FlaA SVR
ST-21	271	2	1	1	3	2	1	5	98	11	8	24	1,2,4^c^,8,10	1,10
ST-45	93	4	7	10	4	1	7	1	48	31	17	44	6, 7, 9, 12, 15, 21, 27, 33, 38, 42, 45, 55, 57, 60	1, 2, 5, 8, 9, 12, 18, 27
ST-206	60	2	21	5	37	2	1	5	37	3	8	9	1,4^c^	1,11
ST-61	57	1	4	2	2	6	3	17	19	2	9	5	4^c^	6,13,14
ST-48	54	2	4	1	2	7	1	5	22	4	5	7	4^c^	1,4
ST-257	46	9	2	4	62	4	5	6	21	4	3	4	11	12
ST-353	29	7	17	5	2	10	3	6	24	9	8	14	3,11,37	2,11
ST-42	22	1	2	3	4	5	9	3	7	5	4	5	23,36,23/36	9
ST-403	20	10	27	16	19	10	5	7	12	5	6	7	35	35
ST-52	18	9	25	2	10	22	3	6	11	7	3	7	5,11	4
ST-177	17	17	2	8	5	8	2	4	17	5	13	7	-	-
ST-354	13	8	10	2	2	11	12	6	4	5	7	8	53	1,20,33
ST-22	12	1	3	6	4	3	3	3	5	2	3	3	19	3
ST-433	11	2	59	4	38	17	12	35	11	6	3	9	1,53	-
ST-362	9	1	2	49	4	11	66	8	1	1	1	1	41	3
ST-179	10	1	6	7	2	40	32	3	9	2	2	3	-	-
ST-49	6	3	1	5	17	11	11	6	2	2	2	2	18	11

^a^ST, sequence type; HS, heat stable; SVR, short variable region; -, no antigen variants were associated that were statistically significant (p>0.001), but isolates were antigenically varied. ^b^HS 4 complex serotypes (4c) were counted as one serotype. ^c^Antigen variants that were significantly associated with a particular clonal complex by the chi-square test. Isolates containing other antigen variants were also found in each complex, as were isolates which were nontypable by HS serotyping.

 Of the 17 clonal complexes, 6 had been described previously (ST-21, ST-45, ST-22, ST-179, ST-177, and ST-52 complexes) (13), and the others (ST-206, ST-61, ST-48, ST-257, ST-353, ST-42, ST-403, ST-52, ST-354, ST-433, and ST-49 complexes) are described here for the first time (Table 2). As a result of the larger dataset and additional analysis techniques used in our study, several STs that had been provisionally described as members of ST-17, ST-65, ST-125, and ST-51 complexes (13) were shown to be members of ST-257, ST-433, ST-206, and ST-353 complexes, respectively, and the provisional names were discarded.

 In 14 of the 17 clonal complexes, the central genotype was distinct from that of any other complex. The three exceptions were ST-21 complex, ST-48 complex, and ST-206 complex, which shared combinations of identical alleles at four of the seven loci (Table 2). These clonal complexes were described as separate entities with their own founder genotype for two reasons. First, each complex had an abundant central genotype, which was present in multiple isolates of the collection, and second, each central genotype had many single-, double-, and triple-locus variants. Isolates belonging to each of these three clonal complexes differed in their association with isolation source. Members of the ST-48 complex were isolated predominantly from human disease, cattle, and sand from beaches; members of the ST-21 and ST-206 complexes were also obtained from poultry (Figure).

**Figure:**
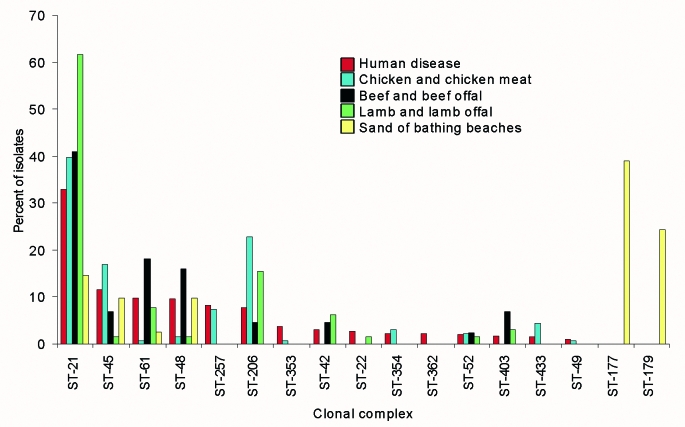
Frequency distribution of *Campylobacter jejuni* clonal complexes isolated from different sources.

### Variation in Cell-Surface Components

Among the 814 isolates were 46 HS antigen serotypes and 64 FlaA SVR amino acid sequence variants in a total of 215 unique combinations. At the nucleotide sequence level, 221 distinct *flaA* SVR sequences were present. The *d_N_* (nonsynonymous base substitutions) to *d_S_* (synonymous base substitutions) ratio was 0.608 for the 321-bp sequence encompassing the SVR: this ratio is comparable with levels ranging from 0.036 to 0.071 for the seven housekeeping gene loci used in MLST (13).

Most of the clonal complexes contained multiple STs, HS serotypes, and FlaA SVR amino acid sequence variants (Table 2). In particular, the two most common complexes among the human disease isolates, ST-21 complex and ST-45 complex, had 24 and 43 combinations of HS serotype and FlaA SVR variant (Tables 2,3). For 15 of the 17 clonal complexes, a significant association (p<0.001) with at least one surface component variant was observed (Table 2), but particular variants generally occurred in more than one clonal complex. Only three of the complexes were associated with a single HS serotype, including an association of ST-22 complex with HS serotype 19, ST-362 complex with HS serotype 41, and ST-257 complex with HS serotype 11 (Table 2). Other complexes were associated with isolates of cross-reactive or likely phase variant serotypes (22,28), including three of the most frequently represented clonal complexes in the isolate collection (ST-206, ST-48, and ST-61 complexes), which contained isolates expressing the following members of the HS 4 serotype 4 complex: 4, 13, 16, 43, 50, and 65. Serotypes 23 and 36, which are highly cross reactive and structurally unique among the HS serotyping antigens (29,30), were only present among isolates belonging to ST-42 complex. Serotypes 27 and 57, which may be expressed by the same isolate at different times (31), were expressed by isolates assigned to the ST-45 complex (Table 3). The isolates assigned to the remaining nine complexes were either associated (p<0.001) with more than one HS serotype (for example, ST-21 complex with HS serotypes 1, 2, and 4 complex) or deemed too small for meaningful statistical analysis (e.g., ST-179 complex contained 10 isolates and appeared to be associated with HS serotypes 2 and 5, and ST-49 complex contained six isolates and was associated with HS serotype 18). The ST-45 complex contained 23 different HS serotypes among its 93 isolates (Table 3), 14 of which were found only in isolates belonging to the ST-45 complex.

**Table 3:** Combinations of cell-surface components associated with ST-21 complex and ST-45 complex

Clonal complex	HS^a^ serotype	FlaA SVR variant(s)
ST-21	1	1,2,8,10,11,46
	2	1,4,5,8,10
	4c	1,4,5,11
	5	1
	8,17	1
	9	1
	10	5,8,10
	11	1
	23	10
	68	1
ST-45	2	12
	3	2
	4c	1,5,9
	5	2
	6	5,8,9,18
	7	5,77
	8	5
	9	1,22
	9,37	12
	12	1
	15	1,24,27
	21	12
	27	5
	33	26,27
	38	1,5
	40	1
	42	1,2,27
	44	5
	45	1,23,24
	53	2
	55	2,27,47
	57	2,5,32
	60	1,2

^a^ HS, heat stable; SVR, short variable region.

 Some of the FlaA SVR variants were associated with particular clonal complexes, for example, variant 12 with ST-257 complex; variants 6,13, and 14 with ST-61 complex; variant 3 with ST-22 and ST-362 complexes; and variant 9 with ST-42 complex. Among the 31 isolates with unique STs, 11 SVR variants were present; 5 were unique. Variant 1 was the most common of these, occurring 13 times among these isolates. Certain HS serotype and SVR variants occurred together to a significant degree (p<0.001, chi-square test), for example, variant 10 with HS serotype 2 and variant 8 with HS serotype 10. Many other combinations were also found but were present in insufficient numbers for statistical analysis. Clonal complexes with the same HS serotype or types did not necessarily share the same FlaA SVR variant or variants. For example, the isolates among ST-48 and ST-61 complexes both belonged to the HS serotype 4 complex but contained different SVR sequences.

### Clonal Complex and Isolation Source

 These results suggest differences in the frequency distribution by isolation source of isolates belonging to particular clonal complexes. The isolates assigned to the ST-21 complex were found in all sources, ranging from 32% of all isolates from human disease to >60% of all isolates from lambs; however, the ST-177 and ST-179 complexes were present only in isolates from beach sand, and ST-257 was present only in the isolates from human disease or chickens. The data also strongly suggest a nonrandom source distribution among the other clonal complexes; for example, ST-61 and ST-48 complexes appeared to be overrepresented in isolates from cattle and beef offal (Figure).

## Discussion

 The Kauffman-White scheme for the typing of *Salmonella enterica* provided a paradigm for the characterization of bacterial pathogens for much of the last century (32). The success of this scheme was largely a consequence of the clonal population structure of this organism, resulting in a tree-like phylogeny, which can be readily reconstructed from any of a number of characteristics (33,34). Variation in the immunologic reactivity of *S. enterica* surface polysaccharides proved to be a convenient way of identifying individual clones, permitting the rapid establishment of the epidemiology of this organism and enabling its spread to be monitored and contained. Similar serologic typing schemes have been developed for other bacterial pathogens, including *C. jejuni* and *C. coli* (11); however, a number of these bacteria, such as *C. jejuni*, engage in lateral gene transfer more frequently than salmonellae (13,17,35). These bacteria do not have a strongly clonal population structure, and loci encoding individual characteristics, such as flagella and capsular antigens, which are under diversifying selection, move by horizontal genetic exchange at relatively high frequency among *C. jejuni* isolates that do not necessarily share a recent common ancestor (36,37). Consequently, these characteristics do not provide epidemiologic information of the same quality as in salmonellae, which explains many of the difficulties in understanding the spread of *Campylobacter* disease in humans.

 The MLST approach, by indexing the nucleotide sequence variation present at several housekeeping loci (which evolve slowly because they are under stabilizing selection for conservation of metabolic function), provides data that are highly discriminatory but enable the population structure of an organism to be established. These data are not sensitive to the “genome instability” observed in *C. jejuni,* which appears to result largely from chromosome rearrangements and which adversely affects typing techniques such as pulsed-field gel electrophoresis fingerprinting (31,38,39). The sequence data presented here were consistent with the view that *C. jejuni* has a weakly clonal population structure, as suggested by previous genotypic and phenotypic studies (17,21,40). Although the isolate collection examined was diverse, most of the 379 observed STs could be assigned to 17 clonal complexes, 6 of which accounted for most of the human disease isolates from the United Kingdom and the Netherlands. Three of the clonal complexes, ST-21, ST-206, and ST-48, may form a “complex group” of related genotypes, which are widely distributed, perhaps reflecting an ability to colonize a wide range of hosts. This complex group probably corresponds to the grouping of heterogeneous yet related isolates, previously referred to as the HS serotype 1, 2, and 4 complex (40–43).

When the level of *flaA* SVR allelic diversity was assessed at the amino-acid level, a degree of discrimination between isolates was obtained comparable with that obtained with clonal complexes. Most isolates contained 1 of 15 SVR variant types (data not shown), and the number of clonal complexes identified was 17. In contrast, at the nucleotide sequence level the allelic diversity of the SVR region was so great that it was less useful for population analysis. However, the nucleotide sequences of genes under diversifying selection are useful when discriminating between related isolates, for example, in distinguishing outbreak strains.

The combination of data for the two cell-surface components, HS serotype and FlaA SVR variant, provided less discrimination than ST, and—unlike the STs—these combinations could not be categorized into larger groups. Some correspondence between the HS serotype/FlaA SVR variant type and clonal complex was observed; for example, all the isolates assigned to the ST-22 complex contained only isolates of HS serotype 19 and FlaA SVR variant 3. However, in general the variation of these molecules was an inconsistent indicator of clonal complex. Such observations have been made previously, with poor correlation detected between the HS antigens and the heat-labile (flagella) antigens (40). Many distinct combinations of HS serotype and FlaA SVR variant type occurred among the isolates assigned to the largest clonal complexes, for example, ST-21 and ST-45 complexes (Table 3), which together accounted for 49% (244/501) of the human disease isolates. A degree of overlap in the HS serotype and SVR variants was also present in a number of the clonal complexes.

 The assignment of the isolates by ST and clonal complex permitted comparisons among isolates obtained from diverse sources. The fact that this classification was possible demonstrated that there was structure to the genetic diversity of *C. jejuni*. Such structure may be imposed in recombining populations by a number of forces, including clonal expansion, niche adaptation, geographic isolation, and host immune selection (44,45). An unambiguous resolution of the evolutionary forces that shape *C. jejuni* populations will only be possible when representative samples of natural populations of this organism, i.e., those present in wild and domestic animals, are tested by multilocus nucleotide sequence-based techniques such as MLST. However, the data presented here are consistent with the view that the clonal complexes may be maintained by niche adaptation, as a number of clonal complexes were overrepresented in particular isolation sources. The ST-45 and ST-257 complexes predominantly contained isolates from human disease and poultry; the ST-61 and ST-42 complexes mainly comprised isolates from human disease, cattle, and sheep; and the ST-177 and ST-179 complexes exclusively contained isolates from beach sand (Figure).

 Immune selection imposed by the host may also play a role in defining the clonal complexes that are uniform in their combinations of HS serotype and FlaA SVR variant: all members of the ST-257 complex had HS serotype 11 and SVR variant 12, and all members of the ST-22 complex contained isolates of HS serotype 19 and SVR variant 3. This finding could also reflect niche adaptation if these surface components were important in the colonization of particular host species. The medical importance of such association is that the ST-22 complex is overrepresented among isolates associated with Guillain-Barré and Miller Fisher syndromes (21), probably on the basis of the lipopolysaccharide antigens that it expresses (46). However, other clonal complexes were more antigenically heterogeneous; thus, a complicating factor in exploring these relationships for all *C. jejuni* may be the organism’s ability to colonize multiple hosts and its exposure to many different immune responses.

 A further advantage of MLST data is that they can be used to examine bacterial species definitions more closely. Our data were consistent with the suggestion that the distinctions between different *Campylobacter* species may be complicated by interspecies genetic exchange (13). The *uncA* allele (*uncA-17*) that defined ST-61, the central genotype of fourth-largest clonal complex in this sample, was highly divergent from all other *uncA* alleles and likely to have originated in another species, perhaps *C. coli*. Ten other STs within the data set contained from one to six divergent alleles that probably originated from other *Campylobacter* species.

 MLST data will enable the appropriate choice of representative isolates for whole genome analyses and studies of animal models. Comparisons of the members of complexes that are never or rarely associated with human disease, examples of which may include the ST-177 and ST-179 complexes reported here, will provide valuable comparisons with isolates from complexes often associated with human disease. Since MLST data are directly comparable and the amount of data available will expand over time, this method should be enable the questions of *Campylobacter* epidemiology to be addressed by the scientific community more easily.

Our study demonstrated the high throughput and cost-effectiveness achievable with nucleotide sequence-based techniques such as MLST: the 814 isolates were characterized at a consumables cost (i.e., cost of supplies, excluding time and labor) of approximately $37 per isolate. The data presented here indicate the likely extent of diversity of *C. jejuni* isolates from humans, permitting investigations to be designed that will determine the relative importance of the food chain—and its individual components—as sources of human *Campylobacter* infection.
